# An Autocollimator Axial Measurement Method Based on the Strapdown Inertial Navigation System

**DOI:** 10.3390/s24082590

**Published:** 2024-04-18

**Authors:** Wenjia Ma, Jianrong Li, Shaojin Liu, Yan Han, Xu Liu, Zhiqian Wang, Changhong Jiang

**Affiliations:** 1 Changchun Institute of Optics, Fine Mechanics and Physics, Chinese Academy of Sciences, Changchun 130033, China; mawenjia21@mails.ucas.ac.cn (W.M.); lijianrong@ciomp.ac.cn (J.L.); liusj@ciomp.ac.cn (S.L.); hanyan23@mails.ucas.ac.cn (Y.H.); liuxu223@mails.ucas.ac.cn (X.L.); 2 University of Chinese Academy of Sciences, Beijing 100049, China; 3 School of Electrical and Electronic Engineering, Changchun University of Technology, Changchun 130012, China

**Keywords:** axis measurement, data fusion, autocollimation, SINS, computational measurement

## Abstract

Autocollimators are widely used optical axis-measuring tools, but their measurement errors increase significantly when measuring under non-leveled conditions and they have a limited measurement range due to the limitations of the measurement principle. To realize axis measurement under non-leveled conditions, this paper proposes an autocollimator axis measurement method based on the strapdown inertial navigation system (SINS). First, the measurement model of the system was established. This model applies the SINS to measure the change in attitude of the autocollimator. The autocollimator was then applied to measure the angular relationship between the measured axis and its own axis, based on which the angular relationship of the axis was measured via computation through signal processing and data fusion in a multi-sensor system. After analyzing the measurement errors of the system model, the Monte Carlo method was applied to carry out a simulation analysis. This showed that the majority of the measurement errors were within ±0.002° and the overall measurement accuracy was within ±0.006°. Tests using equipment with the same parameters as those used in the simulation analysis showed that the majority of the measurement errors were within ±0.004° and the overall error was within ±0.006°, which is consistent with the simulation results. This analysis proves that this method solves the problem of the autocollimator being unable to measure the axis under non-leveled conditions and meets the needs of axis measurement with the application of autocollimators under a moving base.

## 1. Introduction

Axis measurement is an essential method for determining relative position and attitude [1,2], and it is widely used in industrial production, military operations, aerospace, and other fields. It is also employed in scientific research, including in straightness calibration [3], photon energy detection [4], and surface measurement [5]. In the aspect of axis angle measurement, it can be divided into mechanical methods, electromagnetic methods, optical methods, and inertial methods [6]. Among them, the mechanical and electromagnetic methods are more mature and less expensive to measure. However, the accuracy of electrical methods is easily affected by the environment, while mechanical methods are mostly contact measurements, which are limited in many fields. Optical measurement is a non-contact measurement method with high accuracy and sensitivity, which is widely used.

As a type of optical axis-measuring equipment, autocollimators benefit from advantages such as a high measuring accuracy, wide range, and non-contact measurement. The working principle of autocollimator is to use the orientation of the reflected beam from the target for pose calculation, which can realize precise single- and multi-axis angle measurements.

As technology has developed, so have autocollimators, from the traditional optical autocollimators to photoelectric autocollimators. The latter replaces the human eye with sensors such as charge-coupled devices (CCDs), quadrant photodiodes (QPDs), position sensitive detectors (PSDs), or complementary metal oxide semiconductors (CMOSs) for measurement, which improves the resolution and measurement accuracy [7,8,9,10]. The current research on measurement methods based on autocollimators mainly includes several aspects such as improving the measurement accuracy, increasing the measurement range, and increasing the number of measurement targets. For example, the use of new sensors improves the measurement accuracy [11,12,13], the design of the optical system improves the measurement accuracy and range [3,14], and the design of cooperative targeting achieves the measurement of the target’s angle in three directions: yaw, pitch, and roll [15,16].

However, due to the limitation of the autocollimation measurement principle, its measurement range is mainly determined by the field of view of the optical system, the type of light source, and the image sensor, and usually the angular measurement range of the high-precision autocollimator is less than 1°, and the measurement distance is less than 50 m. In addition, in order to ensure measurement accuracy, the autocollimator needs to be roughly leveled with a geodetic coordinate system as a reference before use to avoid causing more significant measurement errors or making measurement impossible. However, in many measurement scenarios, it is impossible to level the autocollimator, which needs to remain stationary during the measurement process, making it impossible to carry out dynamic measurement. Therefore, autocollimators are usually used in the laboratory or after leveling on a stable platform. It is not possible to measure across long distances and on a large scale, such as, for example, in the case of ship installations, where the axes between the upper and lower layers of the hull are measured; in the case of large airplanes, where the axes are calibrated between each of the long-distance axes; or in the case of fast axes measurements of carrier vehicles in an off-site environment.

The principle of the strapdown inertial navigation system (SINS) is based on inertial characteristics; through the fusion data of the internal gyroscope and accelerometer sensors, it can realize accurate measurement of its angle and that of its strapdown equipment relative to the geodetic coordinate system. Accelerometers usually use micro-electromechanical system technology, and the displacement and angle can be inferred by integrating the acceleration in three directions [17,18,19]. Currently, the laser gyro and fiber-optic gyro measurement principles are based on the Sagnac effect, i.e., the beam propagation time slightly differs with rotation, and by measuring the time difference, the rotational speed and direction of an object can be obtained [20,21]. Compared with traditional gyroscopes, they have the advantages of high precision and shock resistance, so they are widely used in navigation systems [22,23].

At present, precision measurement is usually not limited to one kind of equipment, and multi-device cooperative work is one of the hot topics in current research [24,25]. With the improvement of the accuracy of laser gyro, fiber-optic gyro, and micro-electromechanical system (MEMS) inertial guidance technology, as well as the decrease in the cost and volume, inertial guidance has been employed in a large number of applications for the solution of position and navigation under multi-sensors. For example, inertial guidance is usually combined with a global navigation satellite system (GNSS) for integrated navigation [26,27,28], with a Doppler velocity log (DVL) for underwater navigation [29], and with a radar or camera for simultaneous localization and mapping (SLAM) algorithms [30].

Based on SINS characteristics and the defects that mean the autocollimator cannot measure under long-distance, wide-angle, or non-leveled conditions, it is of great practical significance and application value to proposes a non-leveled dynamic axis measurement method based on an SINS and autocollimator.

This paper is organized as follows: In Section 2, the system composition, measurement modeling, and experimental setups are described. Section 3 shows the simulation results and experimental results. Section 4 explains the experimental results, the potential limitations of this study, and how the system could be improved in future work. Section 5 presents the conclusions.

## 2. Methodologies

### 2.1. System Composition

**Figure 1:**
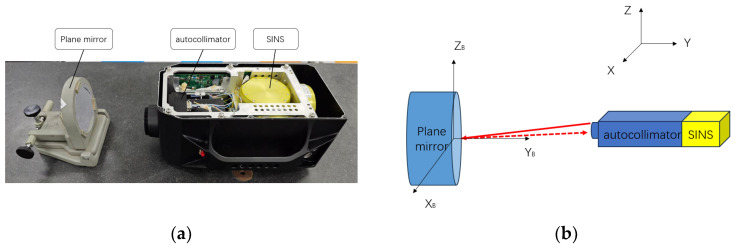
System composition and principle. (**a**) The system includes an autocollimator and a strapdown inertial guide as the measurement equipment and a plane mirror as the target; (**b**) the autocollimator measures the plane mirror’s axis and the SINS measures the geodetic coordinate system. The red arrows represent the return of beam from the collimator after reflection by the plane mirror.

Figure 1a shows the system composition, which includes a dual-axis photoelectric autocollimator and a strapdown inertial guide. The SINS consists of three fiber-optic gyros; it is a customized version purchased by the laboratory. The measurement errors of the inertial guide were within ±0.001° over a short time. The autocollimator consists of an optical system, a light source, and a CMOS sensor. The optical system was made by our lab and is designed for a focal length of 60 mm, an aperture of 25 mm, and a measuring range of 5 m. The model of the CMOS sensor is NOIP1SN5000A, made by ONSEMI, Scottsdale, AZ, USA; the sensor utilizes 4.8 μm × 4.8 μm pixels that support low-noise “pipelined” and “triggered” global shutter readout modes with 2592 × 2048 active pixels, with a plane mirror as the measurement target. The autocollimator has a measurement accuracy of ±0.001° in yaw and pitch under horizontal conditions, theoretically. The SINS has a measurement accuracy of ±0.01° theoretically, and had a measurement accuracy of ±0.001° in a short time proven by tests in the yaw, pitch, and roll directions. The measurement principle is shown in Figure 1b. In the measurement system, the inertial guide is used to measure the angular information between the system and O-XYZ relative to the geodetic coordinate system (i.e., northeast sky coordinate system), and the autocollimator is used to measure the angular relationship between the equipment and the Y_B_ axis of the target being measured (planar mirror). The fusion of the two data points is used to obtain the relationship between the axis of the target being measured relative to the geodetic coordinate system for the yaw and pitch angles.

### 2.2. Measurement Modeling

#### 2.2.1. Coordinate System Establishment

**Figure 2:**
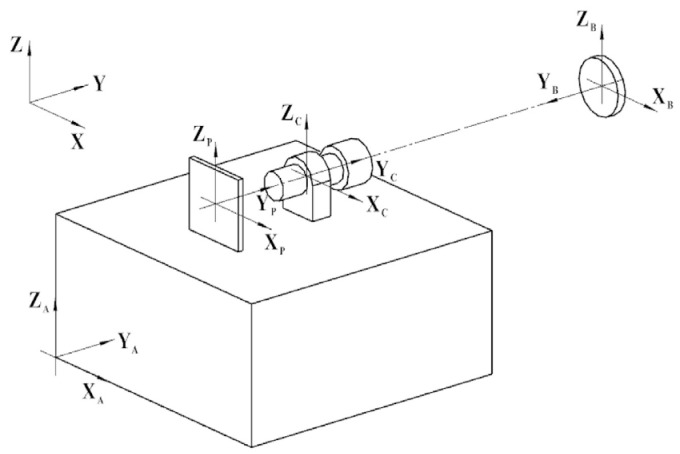
Measurement coordinate system. The measurement model is constructed using the geodetic coordinate system O-XYZ, the SINS coordinate system O-X_A_Y_A_Z_A_, the lens coordinate system O-X_C_Y_C_Z_C_, the camera coordinate system O-X_P_Y_P_Z_P_, and the plane mirror coordinate system O-X_B_Y_B_Z_B_.

The first step in establishing the system measurement model is to establish the measurement coordinate system, as shown in Figure 2, with the plane mirror as the measurement target. O-XYZ is the geodetic coordinate system (i.e., the northeast celestial coordinate system), O-X_A_Y_A_Z_A_ is the SINS coordinate system, O-X_C_Y_C_Z_C_ is the lens coordinate system, O-X_P_Y_P_Z_P_ is the camera coordinate system, and O-X_B_Y_B_Z_B_ is the plane mirror coordinate system. Among them, the inertial coordinate system, the lens coordinate system, and the camera coordinate system are all based on the Earth’s level; the coordinate axes of these three coordinate systems are in the same direction, and the orientation is based on the Y_C_ direction along the optical axis.

#### 2.2.2. Measurement Modeling of the Autocollimator

**Figure 3:**
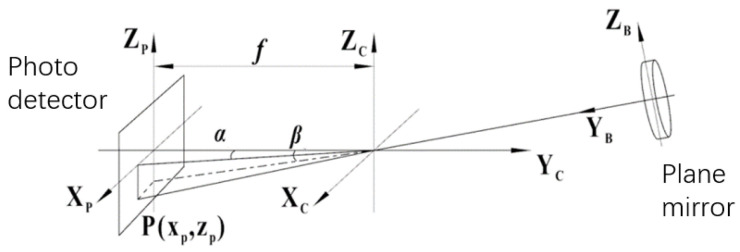
Autocollimator measurement coordinate system. By calculating the position of the reflected point P (*x_p_,z_p_*), the yaw angle α and pitch angle *β* of the plane mirror concerning the autocollimator can be obtained.

First, we consider the measurement model of the autocollimator in a leveled case. Projection along the opposite direction to the X-axis in Figure 2 yields the system shown in Figure 3. For this, we let the focal length of the autocollimator be *f*. The plane mirror reflects the emitted target, point P, to the image plane, point P (*x_p_,z_p_*). Then, the yaw angle α and pitch angle *β* of the plane mirror concerning the autocollimator can be computed using Equation (1).

(1) $$\begin{array}{c} \alpha =\frac{1}{2}*\tan^{-1}\left(\frac{x_{p}}{f}\right)\\ \beta =\frac{1}{2}*\tan^{-1}\left(\frac{z_{p}}{f}\right) \end{array}$$

**Figure 4:**
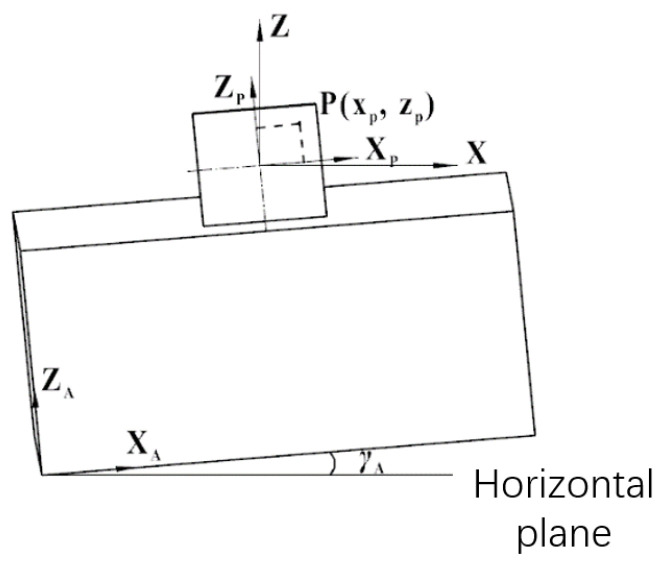
Autocollimator angle measurement under non-leveled conditions. *γ_A_* is the roll angle between the autocollimator and the horizontal plane, which causes measurement errors.

A further analysis of the measurement system under non-leveled dynamic conditions is shown in Figure 4 for the O-XZ planar projection obtained by projecting the measurement coordinate system in Figure 2 along the positive direction of the Y-axis. When the roll angle between the autocollimator and the horizontal plane is *γ_A_*, the target is set to image point P (*x_p_,z_p_*) on the image plane of the camera and converted from the O-X_p_Z_p_ coordinate system to the O-XZ coordinate system. The position coordinate conversion equations are as follows:

(2) $$\begin{array}{c} x=x_{p}*\cos\gamma_{A}-z_{p}*\sin\gamma_{A}\\ z=x_{p}*\sin\gamma_{A}+z_{p}*\cos\gamma_{A} \end{array}$$

Substituting Equation (2) into (1) yields the relative value of the measurement target to the axis of the measurement system in the non-leveled state:

(3) $$\begin{array}{c} \alpha =\frac{1}{2}*\tan^{-1}((x_{p}*\cos\gamma_{A}-z_{p}*\sin\gamma_{A})/f)\\ \beta =\frac{1}{2}*\tan^{-1}((x_{p}*\sin\gamma_{A}+z_{p}*\cos\gamma_{A})/f) \end{array}$$

#### 2.2.3. Measurement Modeling of the SINS

**Figure 5:**
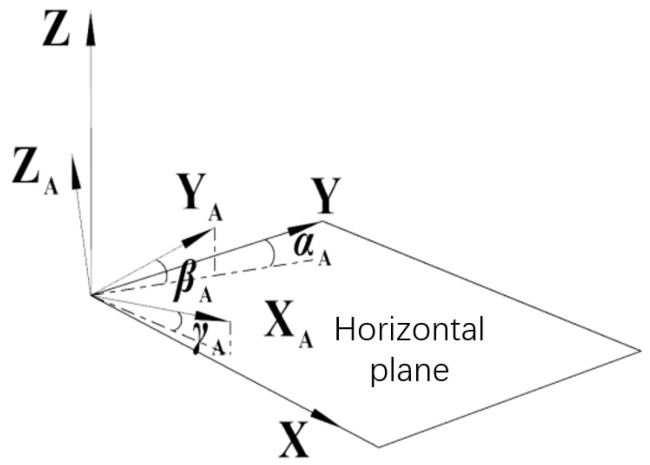
SINS measurement coordinate system. The yaw angle *α_A_*, pitch angle *β_A_*, and roll angle *γ_A_* can be measured using the SINS.

As shown in Figure 5, the inertial guidance measurement angles are defined, respectively, as follows:

1. Yaw angle, *α_A_*: the horizontal angle between the projection of the Y_A_-axis onto the horizontal plane and the actual north direction;
2. Pitch angle, *β_A_*: the angle between the Y_A_-axis in the vertical projection plane and the horizontal plane;
3. Roll angle, *γ_A_*: the angle between the X_A_-axis in the vertical projection plane and the horizontal plane.

Combined with Equation (3), the orientation and pitch angle of the measurement target relative to the geodetic coordinate system can be obtained as follows:

(4) $$\begin{array}{c} \alpha_{0}=\frac{1}{2}*\tan^{-1}((x_{p}*\cos\gamma_{A}-z_{p}*\sin\gamma_{A})/f)+\alpha_{A}\\ \beta_{0}=\frac{1}{2}*\tan^{-1}((x_{p}*\sin\gamma_{A}+z_{p}*\cos\gamma_{A})/f)+\beta_{A} \end{array}$$

### 2.3. Measurement Error Analysis

Based on the measurement model and the calculation formulae provided in Section 2, the sources of errors in the measurement system are further analyzed to prove the validity of the measurement system and its measurement accuracy.

The measurement errors of the system include systematic errors and random errors. Using Equation (4), the systematic errors of the system include the installation errors and focal length errors, and the random errors include the pixel errors and SINS measurement errors.

#### 2.3.1. Installation Errors

Due to the virtual axis of the SINS, it is difficult to achieve parallelism between the coordinate system of the SINS and the autocollimator’s coordinate system through installation and adjustment, which leads to a small deviation between the two coordinate systems. If we let the angle of the autocollimator rolling direction relative to the horizontal plane be *γ_A_*, the angle between the autocollimator rolling axis and the inertial guide rolling axis be *γ*, and the angle between the inertial guide rolling axis and the horizontal plane be *γ_0_*, then we have

(5) $$\gamma_{A}=\gamma_{0}+\gamma$$

Substituting Equation (5) into (4), we obtain

(6) $$\begin{array}{c} \alpha_{0}=\frac{1}{2}*\tan^{-1}((x_{p}*\cos\left(\gamma_{0}+\gamma \right)-z_{p}*\sin\left(\gamma_{0}+\gamma \right))/f)+\alpha A\\ \beta_{0}=\frac{1}{2}*\tan^{-1}((x_{p}*\sin\left(\gamma_{0}+\gamma \right)+z_{p}*\cos\left(\gamma_{0}+\gamma \right))/f)+\beta A \end{array}$$

From Equation (6), the roll angle *γ* between the SINS and the autocollimator affects the measurement results, and this error cannot be eliminated in the relative measurement.

This systematic error can usually be measured and corrected. The measurement of angle *γ* is usually realized through methods such as optical–mechanical calibration. However, the SINS cannot be measured accurately, because of its virtual axis. According to engineering experience, this measurement error can be controlled at about 10″, so the cross-roll error in the subsequent simulation is taken as *γ* = ±0.003°.

#### 2.3.2. Focal Length Errors

The lens’s focal length is usually determined by the optical design, but manufacturing and mounting errors will cause the focal length to be inconsistent with the design value. According to engineering experience, the actual focal length of the lens can usually be controlled within 0.1–0.5% of the theoretical value. After calibration, the actual focal length value can be controlled within 0.1% [31]. In this study, the theoretical focal length of the self-collimator is 60 mm and the actual value after calibration measurement is 59.64 mm. Accordingly, the following simulation takes the focal length error value as ±0.05 mm.

#### 2.3.3. Pixel Errors

In Equation (5), *x_p_* and *z_p_* are the coordinates of the target’s imaging position on the camera at the time of measurement. The errors in *x_p_* and *z_p_* are determined by the resolution of the camera image element. According to research on the pixel subdivision algorithm, the pixel errors can usually be reduced to within 0.1 pixels using differential calculation and other methods [32]. The actual camera pixel resolution used in this system is 4.8 μm. In order to ensure that the simulation was consistent with the actual test, the pixel error value was taken to be 0.5 pixels in the simulation process, which is 2.4 μm.

#### 2.3.4. SINS Measurement Errors

From Section 2.2.2, the measurement error of the SINS is determined by its measurement accuracy. This system uses the fiber-optic inertial guide for measurement. For the measurement system in this study, the measurement is usually completed in a short period of time in a single power-up, so the effects of system errors can be ignored. The random errors of fiber-optic inertial guides include zero bias and noise. However, random errors usually have a negligible effect on the measurement results when measuring in a short period of time. After testing the inertial guide used in the system, it was proven that the measurement errors of this inertial guide were within ±0.001° over a short period of time, so this value was taken for the simulation.

### 2.4. Experiments

Experimental validation was carried out using laboratory equipment to verify the validity of the measurement model and simulation results. The instrumentation used in this testing included the SINS, an autocollimator, a roll adjustment stage, a plane mirror with a 2D adjustment stage, and a parallel light tube for measurements with the following accuracies:

- The installation error was taken as *γ* = ±0.003°;
- The focal length error was taken as ±0.05 mm;
- The CMOS sensor measurement error was taken as ±0.1 pixel;
- The SINS measurement error was taken as ±0.001° in yaw, pitch, and roll;
- The parallel light pipes had a measurement accuracy of ±0.2″ in yaw and pitch;
- The roll adjustment table had a ±15° adjustment range.

**Figure 6:**
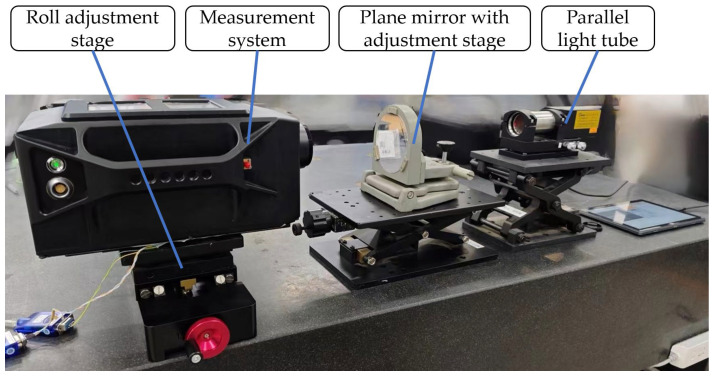
Experimental setup, including a measurement system, a plane mirror (measurement target), and a parallel optical tube to measure the true value of the amount of change in the target. The measurement accuracy of the system is verified by changing the angle of the plane mirror.

The experimental setup is shown in Figure 6. The experimental procedure was as follows: The measuring system (SINS with the autocollimator) was placed on the roll adjustment stage, and the plane mirror with the 2D adjustment stage was placed between the measuring system and the parallel light tube. Adjusting the 2D adjustment table changed the plane mirror’s axis, and its axis changes were monitored with the parallel light tube. Measurement started from the position when the SINS was horizontal, and the range of roll adjustment was ±10°, with an interval of 2° between each adjustment. After each set of the system’s roll values was adjusted, its yaw and pitch values were adjusted using the initial angle of the plane mirror as a reference. Each yaw and pitch adjustment interval was 0.2°, with a range of ±1°. The yaw and pitch values of each set of the plane mirror adjustments were recorded, as well as the coordinate points of the image obtained from the autocollimator in the image plane (*x_p_,z_p_*). A total of 121 sets of corresponding data values were obtained.

## 3. Results

### 3.1. Simulation Results

To ensure the accuracy and validity of the measurement model presented in Section 2.2, as well as the error analysis provided in Section 2.3, a Monte Carlo analysis based on Equation (6) was conducted. The Monte Carlo method, also known as the statistical test method, is a numerical simulation technique that focuses on probabilistic phenomena. Its fundamental principle is random sampling. By constructing a probabilistic model that closely represents the measurement system’s performance and running random trials, the simulation can replicate the system’s random measurement characteristics. The simulation results can be considered as actual measurement outcomes when the number of simulations is large enough. Substituting the four measurement error values from the error analysis in Section 2.3, all of the parameters used in the simulation were the same as those used in the experiments, specifically including the following:

- The installation error was taken as *γ* = ±0.003°;
- The focal length error was taken as ±0.05 mm;
- The CMOS sensor measurement error was taken as ±0.1 pixel;
- The SINS measurement error was taken as ±0.001° in yaw, pitch, and roll.

The specific calculation steps were as follows:

1. Randomly generate an initial set of truth data, including the measurement of the system’s roll angle (within ±5°) and the yaw and pitch angles of the plane mirror axis measured with the autocollimator (within ±4.5°);
2. Based on the generated truth data, back-project the theoretical truth data measured with the sensor, and randomly add the error data in Section 3.1 to them as the measurement data of the sensor;
3. Substituting the sensor measurement data into Equation (4), recalculate the yaw and pitch angles of the plane mirror as measurements;
4. Calculate the difference between the measured data and the true value data as the measurement error.

**Figure 7:**
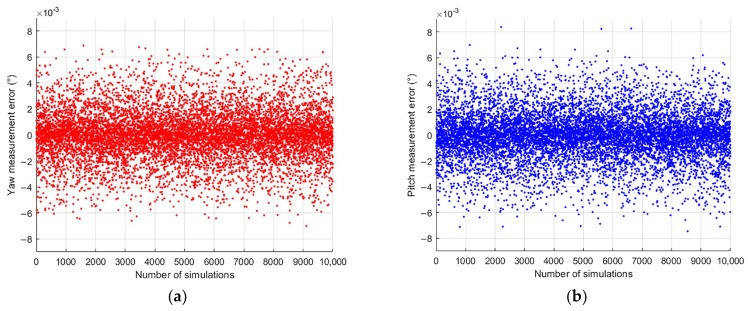

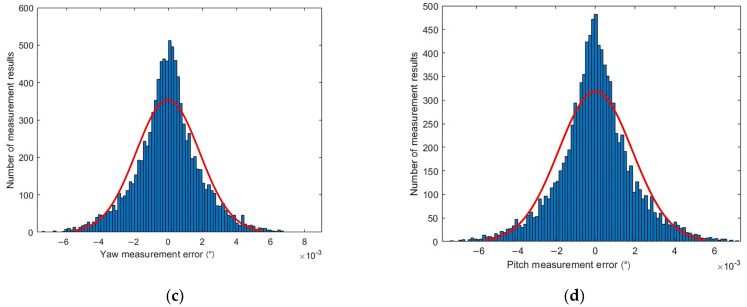
Simulation results. The red line refers to the probability distribution curve fitted to the simulated data. (**a**) Yaw error scatter plot; (**b**) pitch error scatter plot; (**c**) yaw error histogram; (**d**) pitch error histogram.

Scatter plots of the measurement errors of the system and the distribution of the errors when repeating the calculation 10,000 times are shown in Figure 7. The figures show that the system’s measurement errors are overwhelmingly within the range of ±0.002° and the overall measurement accuracy is within the range of ±0.006°. The mean square deviations of the yaw and pitch errors are σ__yaw_ = 0.0020° (1σ) and σ__pitch_ = 0.0019° (1σ).

### 3.2. Experimental Results

The data measured in Section 2.4 are listed in Table 1. The measurement group 0 includes the initial yaw and pitch values of the mirror, as well as the position of the reflected target. For measurement groups 1 to 5, we sequentially increased the yaw and pitch of the plane mirror by about 0.2° in the same direction, using measurement group 0 as a reference. For measurement groups 6 to 10, we sequentially increased the yaw and pitch of the plane mirror by about 0.2° in the other direction, using measurement group 0 as a reference. The exact amount of change was measured by means of a parallel light tube, and the corresponding *x_p_* and *z_p_* values were also recorded.

We substituted the *x_p_* and *z_p_* data in Table 1 into Equation (6) to calculate the measurement values of the mirror’s change in yaw and pitch. The measurement group 0 was also used as the initial value in these calculations, and the measured values were compared with the true values to obtain the errors. From these calculations, the measurement errors are shown in Table 2, and each measurement error matches those of the measurement groups in Table 1.

**Figure 8:**
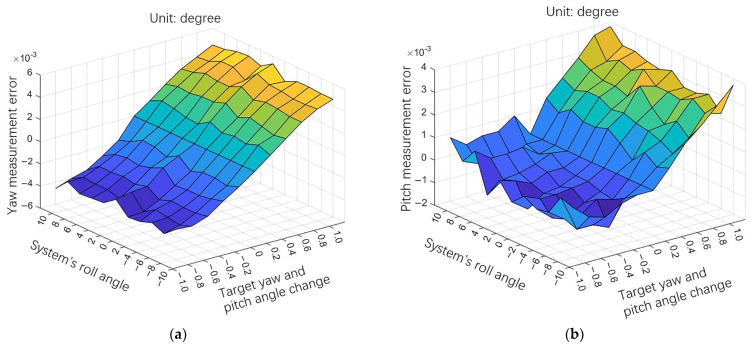
Experimental error distribution. Different color represents the amount of the surface in the z-axis direction. (**a**) Yaw error distribution; (**b**) pitch error distribution.

Analyzing the measurement errors in Table 2 and Figure 8 reveals the following:

When the system measures within ±10° of the roll angle, most of the measurement errors are within ±0.004° and the overall error is within ±0.006°. The root mean square (RMS) values of the measurement errors are σ__yaw_ = 0.003° (1σ) and σ__pitch_ = 0.0018° (1σ). The measurement consistency is good, which is consistent with the simulation results.

## 4. Discussion

Through Monte Carlo simulation of this measurement model, the variance of the yaw and pitch is obtained as σ_yaw = 0.0020° (1σ) and σ_pitch = 0.0019° (1σ) after running the simulation 10,000 times. It can be deduced from the simulation results that the measurement error of this system is stable and consistent for the target yaw and pitch angles.

As can be seen from the experimental results, when the system roll angle is unchanged and the angle of the measurement target changes, the measurement error increases with the change in angle of the measurement target, and the sign of the measurement error is related to the sign of the change in angle of the target. Also, within a specific range, the system’s measurement error does not change due to the change in roll angle.

Comparing the experimental results with the simulation results, it can be seen that the yaw measurement error of the experimental results is larger than that of the simulation results. The reason for these different results may be the fact that the measurement group is too small to reflect the actual measurement capability of the system, or the fact that the CMOS sensor in the autocollimator does not coincide with the axis of the optical system when it is installed, or other reasons, which need to be further explored.

For axis measurements under non-leveled dynamic conditions, the accurate measurement of angles is usually achieved by optimizing the measurement environment, e.g., by designing vibration damping and leveling planes. This study started without optimizing the measurement equipment, which has an advantage in terms of preparation time, volume, and weight, although the accuracy is slightly lower in comparison. This measurement accuracy can still be suitable for vehicle-related axis measurement, aircraft axis measurement target calibration, and naval weapon axis measurement. Since the work presented in this paper involves only a measurement model and its validation under laboratory conditions, subsequent field tests need to be carried out to explore its engineering practicality further.

In this study, the data between the two sensors were only calculated and processed using the measurement model, but there may have been a time delay in the two sensors, which could have led to a decrease in the measurement accuracy due to the unsynchronized data when measuring on a dynamic platform; so, further exploration of data fusion and synchronization methods is required subsequently.

## 5. Conclusions

In order to realize axis measurement under non-leveled dynamic conditions using an autocollimator and extend the measuring range, an autocollimator axis measurement method based on the SINS is proposed. This article demonstrates the system model and measurement calculations, simulation analyses, and experimental verification of the model were carried out. The latter demonstrated that the majority of the method’s measurement errors were within ±0.002° and the overall measurement error was within ±0.006°. The measurement system was tested over a roll angle range of ±10°, showing that most of the measurement errors were within ±0.004° and the overall measurement error was within ±0.006°. This was consistent with the simulation results, showing a good measurement consistency.

Our system has certain advantages over other measurement methods, and the measurement accuracy can be further improved from the point of view of data fusion and synchronization of the two sensors in future work. According to the proposed study, a new measurement method can be provided for axis measurement in the case of a moving base.

## Figures and Tables

**Table 1:** Experimental data.

Roll Angle (°)	Data Type	Measurement Groups										
		0	1	2	3	4	5	6	7	8	9	10
−0.001	Mirror yaw (°)	58.9891	59.1843	59.3862	59.5881	59.7882	59.9858	58.7882	58.5838	58.3834	58.1838	57.9819
	Mirror pitch (°)	90.2941	90.4921	90.6908	90.8952	91.0932	91.2998	90.0947	89.8982	89.6941	89.4959	89.2971
	*x_p_* in CMOS	996.21	1082.25	1171.07	1259.81	1347.67	1434.39	908.08	818.45	730.23	642.92	554.45
	*z_p_* in CMOS	985.64	1072.45	1159.24	1248.72	1334.92	1425.21	898.62	812.91	723.92	637.33	550.85
2.010	Mirror yaw (°)	58.9342	59.1346	59.3338	59.5343	59.7375	59.9371	58.7369	58.5367	58.3347	58.1356	57.9309
	Mirror pitch (°)	90.2945	90.4973	90.6951	90.8961	91.0947	91.2951	90.0933	89.8943	89.6949	89.4949	89.2946
	*x_p_* in CMOS	996.78	1081.68	1166.11	1251.16	1337.37	1421.56	912.98	828.17	742.57	658.29	571.78
	*z_p_* in CMOS	985.57	1077.33	1166.81	1257.67	1347.51	1437.87	894.77	805.01	715.01	624.82	534.54
4.002	Mirror yaw (°)	58.9664	59.1654	59.3642	59.5692	59.7686	59.9669	58.7627	58.5658	58.3646	58.1605	57.9634
	Mirror pitch (°)	90.2976	90.4936	90.6922	90.8951	91.0959	91.2957	90.0921	89.8914	89.6961	89.4982	89.2925
	*x_p_* in CMOS	996.29	1077.64	1158.83	1242.53	1323.64	1404.31	913.21	832.94	750.86	667.69	587.58
	*z_p_* in CMOS	985.66	1077.11	1170.01	1264.51	1358.31	1451.31	889.87	796.47	705.27	613.01	517.77
6.003	Mirror yaw (°)	58.9835	59.1883	59.3879	59.5843	59.7841	59.9892	58.7867	58.5819	58.3824	58.1865	57.9832
	Mirror pitch (°)	90.2931	90.4925	90.6961	90.8969	91.0935	91.2999	90.0967	89.8933	89.6947	89.4956	89.2954
	*x_p_* in CMOS	996.43	1076.82	1154.83	1231.56	1309.67	1389.84	919.34	839.02	761.07	684.71	605.31
	*z_p_* in CMOS	985.58	1081.81	1179.34	1275.66	1370.41	1469.31	891.17	793.71	698.31	603.02	507.11
8.007	Mirror yaw (°)	59.0043	59.2053	59.4065	59.6031	59.8038	60.0064	58.8056	58.6067	58.4061	58.2053	58.0048
	Mirror pitch (°)	90.2923	90.4947	90.6924	90.8946	91.0965	91.2957	90.0921	89.8955	89.6918	89.4951	89.2913
	*x_p_* in CMOS	996.53	1071.88	1147.14	1220.53	1295.46	1371.41	922.08	847.62	772.76	697.43	622.66
	*z_p_* in CMOS	985.35	1085.26	1182.96	1282.49	1382.28	1480.58	886.62	789.21	689.19	592.02	492.45
10.005	Mirror yaw (°)	59.0086	59.2059	59.4079	59.6074	59.8075	60.0061	58.8081	58.6015	58.4021	58.2022	58.0018
	Mirror pitch (°)	90.2878	90.4854	90.6857	90.8844	91.0879	91.2847	90.0821	89.8816	89.6849	89.4802	89.2815
	*x_p_* in CMOS	996.58	1066.86	1139.08	1210.33	1281.35	1352.29	924.97	850.85	779.46	708.71	637.19
	*z_p_* in CMOS	985.35	1085.76	1187.43	1288.21	1391.17	1490.91	882.17	780.19	680.59	577.41	477.05
−2.006	Mirror yaw (°)	58.9537	59.1531	59.3571	59.5554	59.7552	59.9555	58.7547	58.5509	58.3521	58.1568	57.9529
	Mirror pitch (°)	90.2958	90.4984	90.6992	90.8985	91.0972	91.2952	90.0911	89.8948	89.6965	89.4907	89.2906
	*x_p_* in CMOS	996.41	1087.01	1179.91	1270.05	1360.52	1451.51	905.81	813.51	723.12	634.23	542.01
	*z_p_* in CMOS	985.56	1071.19	1155.72	1239.63	1323.51	1406.63	899.65	817.05	733.63	647.11	563.26
−4.004	Mirror yaw (°)	59.0021	59.2047	59.4041	59.6004	59.8015	60.0088	58.8028	58.6031	58.4021	58.2037	58.0012
	Mirror pitch (°)	90.2947	90.4979	90.6974	90.8953	91.0954	91.2957	90.0922	89.8922	89.6928	89.4938	89.2909
	*x_p_* in CMOS	996.21	1091.18	1184.89	1276.97	1371.23	1468.07	902.77	809.02	715.01	622.04	527.32
	*z_p_* in CMOS	985.37	1067.94	1148.86	1229.01	1310.05	1390.91	903.54	822.61	742.01	661.53	579.94
−6.000	Mirror yaw (°)	58.9945	59.1977	59.3999	59.5983	59.7976	59.9975	58.7913	58.5925	58.3916	58.1939	57.9945
	Mirror pitch (°)	90.2922	90.4963	90.6957	90.8931	91.0932	91.2976	90.0955	89.8923	89.6914	89.4948	89.2926
	*x_p_* in CMOS	996.27	1094.52	1191.97	1287.85	1383.99	1480.52	898.61	802.44	705.53	610.31	514.13
	*z_p_* in CMOS	985.32	1064.99	1142.36	1218.89	1296.79	1376.34	909.37	830.68	752.81	676.65	598.34
−8.001	Mirror yaw (°)	58.9714	59.1781	59.3785	59.5754	59.7751	59.9758	58.7727	58.5719	58.3701	58.1722	57.9702
	Mirror pitch (°)	90.2911	90.4971	90.6935	90.8979	91.0926	91.2933	90.0906	89.8917	89.6966	89.4977	89.2969
	*x_p_* in CMOS	996.14	1098.83	1197.91	1296.01	1394.55	1494.01	897.39	798.02	698.53	600.47	500.78
	*z_p_* in CMOS	985.68	1062.31	1135.11	1211.58	1283.52	1358.11	911.36	837.71	765.87	692.09	617.83
−10.006	Mirror yaw (°)	58.9665	59.1671	59.3617	59.5681	59.7611	59.9665	58.7616	58.5667	58.3666	58.1639	57.9635
	Mirror pitch (°)	90.2917	90.4933	90.6942	90.8944	91.0939	91.2957	90.0971	89.8946	89.6902	89.4913	89.2969
	*x_p_* in CMOS	996.36	1098.48	1198.05	1302.56	1401.21	1505.27	893.01	793.38	691.28	588.71	487.71
	*z_p_* in CMOS	985.79	1057.29	1129.02	1199.38	1270.61	1341.91	917.94	845.85	773.25	703.45	635.44

**Table 2:** Measurement errors.

Roll Angle (°)	Data Type	Measurement Groups									
		1	2	3	4	5	6	7	8	9	10
−0.001	Yaw error (°)	0.0016	0.0029	0.0040	0.0048	0.0053	−0.0007	−0.0014	−0.0027	−0.0028	−0.0031
	Pitch error (°)	0.0013	0.0018	0.0028	0.0026	0.0031	−0.0004	−0.0007	−0.0008	−0.0013	−0.0008
2.010	Yaw error (°)	0.0011	0.0021	0.0033	0.0043	0.0042	−0.0016	−0.0025	−0.0034	−0.0041	−0.0042
	Pitch error (°)	0.0009	0.0016	0.0021	0.0026	0.0026	−0.0004	−0.0005	−0.0006	−0.0006	−0.0003
4.002	Yaw error (°)	0.0013	0.0026	0.0036	0.0042	0.0047	−0.0012	−0.0024	−0.0030	−0.0034	−0.0041
	Pitch error (°)	0.0004	0.0015	0.0016	0.0025	0.0026	−0.0006	−0.0009	−0.0013	−0.0012	−0.0006
6.003	Yaw error (°)	0.0011	0.0023	0.0035	0.0040	0.0047	−0.0012	−0.0024	−0.0031	−0.0036	−0.0037
	Pitch error (°)	0.0010	0.0013	0.0020	0.0028	0.0028	−0.0007	−0.0005	−0.0010	−0.0010	−0.0005
8.007	Yaw error (°)	0.0015	0.0020	0.0033	0.0040	0.0046	−0.0014	−0.0022	−0.0031	−0.0038	−0.0042
	Pitch error (°)	0.0006	0.0010	0.0015	0.0024	0.0022	−0.0004	−0.0015	−0.0012	−0.0012	0.0004
10.005	Yaw error (°)	0.0011	0.0022	0.0033	0.0041	0.0046	−0.0010	−0.0019	−0.0029	−0.0034	−0.0033
	Pitch error (°)	0.0008	0.0017	0.0023	0.0030	0.0037	0	−0.0002	−0.0006	−0.0004	0.0002
−2.006	Yaw error (°)	0.0009	0.0025	0.0035	0.0037	0.0046	−0.0013	−0.0019	−0.0030	−0.0039	−0.0039
	Pitch error (°)	0.0011	0.0017	0.0021	0.0031	0.0029	−0.0003	−0.0003	−0.0006	−0.0003	−0.0001
−4.004	Yaw error (°)	0.0010	0.0025	0.0035	0.0044	0.0050	−0.0009	−0.0022	−0.0028	−0.0036	−0.0040
	Pitch error (°)	0.0011	0.0020	0.0023	0.0028	0.0030	0.0001	−0.0002	−0.0004	−0.0005	−0.0010
−6.000	Yaw error (°)	0.0013	0.0023	0.0037	0.0044	0.0049	−0.0008	−0.0020	−0.0029	−0.0036	−0.0041
	Pitch error (°)	0.0014	0.0020	0.0023	0.0030	0.0032	−0.0002	0.0003	0.0002	0.0002	0.0007
−8.001	Yaw error (°)	0.0015	0.0024	0.0033	0.0039	0.0046	−0.0014	−0.0022	−0.0029	−0.0035	−0.0035
	Pitch error (°)	0.0010	0.0018	0.0025	0.0027	0.0032	0	−0.0003	−0.0003	−0.0003	−0.0005
−10.006	Yaw error (°)	0.0010	0.0021	0.0031	0.0038	0.0045	−0.0018	−0.0028	−0.0037	−0.0041	−0.0046
	Pitch error (°)	0.0014	0.0021	0.0028	0.0036	0.0038	0.0010	−0.0005	0.0005	0.0003	0.0008

## Data Availability

The data presented in this study are available on request from the corresponding author.
